# 

**Published:** 1872-02

**Authors:** 


					﻿PUBLISHED MONTHLY, AT $3 PER ANNUM, IN ADVANCE.
J	■
s
* o	I O i
3	SSr
1 Medical and Surgical Journal.!^
S	V	I K
I
(4	I O
o	__
:§
\	‘I;
g.	EDITED BY	i’5‘
'?*
I	J. P. LOGAN, M.D.,	i!
o’	I »
g	'o-,
“	,«<
3-; AV. P. AVESTilOEELAND, SLD., 1
C4 ■
a ■ Professor of the Principles and Practice of Surgery in Atlanta Medical College, g
o'	P
£ '	'®
o	-------------------—	,
^Cbz et Scierbtiaj sedL veritas sine timore, •	ig*
fli	—____________________ - B
©
.	I
S'
I VOLUME IXB-FEBRUARY, 1872.-NUMBER 11.	' t
F-l '	i &
S;	i“
i	a
'	I
gi	-------».♦><..--- I,
g
«	iB
' s
1	I
M
2	ATLANTA, GEORGIA;	?
H	S
,o PLAXTATIOX PUBLISHIXG COMPAXY.
1871.	I ,
EACH NUMBER CONTjM^S SI)n'Y-JFOURJ>AQES,________I
				

